# Extraction of Phenolic and Flavonoid Compounds from Mung Bean (*Vigna radiata* L.) Seed Coat by Pressurized Liquid Extraction

**DOI:** 10.3390/molecules27072085

**Published:** 2022-03-24

**Authors:** Benya Supasatyankul, Maythee Saisriyoot, Utai Klinkesorn, Kittipong Rattanaporn, Sudathip Sae-Tan

**Affiliations:** 1 Department of Food Science and Technology, Faculty of Agro-Industry, Kasetsart University, Bangkok 10900, Thailand; benya.su@ku.th (B.S.); utai.k@ku.th (U.K.); 2 Department of Chemical Engineering, Faculty of Engineering, Kasetsart University, Bangkok 10900, Thailand; fengmts@ku.ac.th; 3 Department of Biotechnology, Faculty of Agro-Industry, Kasetsart University, Bangkok 10900, Thailand; kittipong.r@ku.th

**Keywords:** flavonoid extraction, mung bean, phenolic extraction, pressurized liquid extraction, response surface methodology

## Abstract

Mung bean seed coat (MBC) is a by-product of the mung bean processing industry. It contains a large number of phenolic compounds with therapeutic anti-inflammatory, anti-diabetic and antioxidant properties. This research aimed to investigate the optimum conditions for phenolic and flavonoid extraction from MBC by pressurized liquid extraction (PLE). Response surface methodology (RSM) was used to study the effects of temperature (80–160 °C), pressure (1200–1800 psi) and ethanol concentration (5–95%) on total phenolic content (TPC), total flavonoid content (TFC) and 2,2′-azinobis(3-ethylbenzothiazoline-6-sulfonic acid) scavenging activity (ABTS). Scale-up extraction was also performed. The optimum conditions for extraction were 160 °C, 1300 psi and 50% ethanol. Under optimum conditions, the TPC was 55.27 ± 1.14 mg gallic acid equivalent (GAE)/g MBC, TFC was 34.04 ± 0.72 mg catechin equivalent (CE)/g MBC and ABTS scavenging activity was 195.05 ± 2.29 mg trolox equivalent (TE)/g MBC. The TFC and ABTS scavenging activity of the extracts obtained at the pilot scale (10 L) was not significantly different from the laboratory scale, while TPC was significantly increased. The freeze-dried MBC extract contained vitexin and isovitexin 130.53 ± 17.89, 21.21 ± 3.22 mg/g extract, respectively. In conclusion, PLE was able to extract phenolics, flavonoids with ABTS scavenging activity from MBC with the prospect for future scale-up for food industry.

## 1. Introduction

Mung bean (*Vigna radiata* L.) is a pulse crop widely cultivated and consumed in Asia, India and the warmer part of Europe and America, with a short growth cycle around 2–3 months [1]. Mung bean is a rich source of proteins, essential amino acids, complex carbohydrates, vitamins and minerals and it is easy to be digested [2]. Mung bean is also well known for a large number of bioactive compounds including protein, phenolic and flavonoid compounds with various health benefits [3]. The majority of by-products from the mung bean industry is mung bean seed coat and it is usually discarded. While phenolic and flavonoid compounds were reported to be abundant in mung bean seed coat for 84.2% and 83.9%, respectively [4]. Many biological activities of mung bean seed coat extract were reported. A recent study showed that mung bean seed coat water extract exerted anti-inflammatory effects by inhibiting NF-κB activation via inhibition of TAK1 phosphorylation and IκBα degradation [5]. Mung bean seed coat water extract also showed antioxidant activity [6]. While ethanolic extract of mung bean seed coat inhibited α-glucosidase activity and exerted anti-diabetic activity in *db*/*db* mice [7]. The proposed bioactive compounds in mung bean seed coat were vitexin and isovitexin [5]. Total phenolic content in mung bean extract is ranging from 2.03 to 79.65 mg GAE/g extract, depending on the method of extraction [8,9,10]. Therefore, the study on the extraction method and optimum conditions is necessary.

Conventional extraction methods for polyphenols, phenolics and flavonoids from plant materials are maceration, hydro-distillation, agitated solvent extraction and Soxhlet extraction [11]. Recently, many innovative extraction methods have been introduced to increase the efficacy of extraction including ultrasound-assisted extraction (UAE), microwave-assisted extraction (MAE) and pressurized liquid extraction (PLE). Santos et al. compared the efficacy of α-bisabolol extraction between Soxhlet extraction and PLE [12]. The results showed that the amount of α-bisabolol from both methods was not significantly different. However, PLE used only 20 min, while Soxhlet extraction used 360 min for extraction. PLE was compared with UAE in extracting phenolic compounds from the residues of *Rubus fruticosus*, *Vaccinium myrtillus* and *Eugenia brasiliensis* [13]. The results showed that PLE gave a higher yield of phenolic compounds compared to UAE. PLE was also more efficient in the extraction of terpenes, fatty acids and vitamin E from *Piper gaudichaudianum* Kunth in terms of yield and time [14]. The efficacy of PLE and MAE in extracting phenolic compounds from *Moringa oleifera* leaves was compared by Barriada-Pereira et al. [15]. The results showed that TPC and TFC from PLE were higher than those from MAE. This indicated that PLE was a potential extraction method to increase the efficacy of phenolic compounds from mung bean seed coat.

Many factors play a role in extraction. The efficiency of PLE depends on the extraction temperature, pressure, time, solvent and the ratio between extraction solvent and sample [16]. The present study was designated to investigate the effects of extraction temperature, pressure and concentration of ethanol on TPC, TFC and ABTS of mung bean seed coat. The main goal of the present study was to optimize, validate and scale up the extraction of mung bean seed coat using PLE.

## 2. Results

### 2.1. Comparison of Soxhlet and PLE Extraction

**Figure 1:**
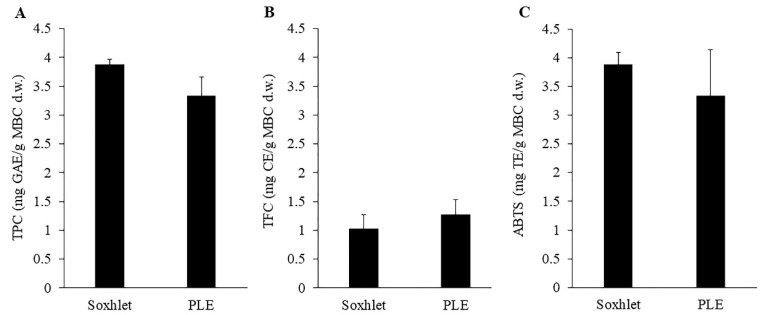
Total phenolic content (**A**), total flavonoid content (**B**) and ABTS (**C**) of MBC extract obtained by Soxhlet extraction (80 °C and 95% ethanol) and PLE (80 °C, 1500 psi and 95% ethanol). There was no significant difference between methods on those response variables.

Considering the high number of bioactive compounds and therapeutic properties of MBC, the extraction of phenolic and flavonoid compounds from MBC was interesting. The total content of bioactive compounds depends on many factors including the extraction method [17]. Here, MBC was extracted with Soxhlet and PLE extraction methods. The TPC, TFC and ABTS of the MBC extract using two extraction methods were shown and compared in Figure 1. The results showed no significant difference between TPC, TFC and ABTS of the extracted solutions from Soxhlet and PLE. About the extraction time, the PLE method spent only 10 min for extraction, while the Soxhlet method spent 6 h for extraction. Both extraction methods were carried out using 95% ethanol at 80 °C, however, the pressure of 1500 psi was applied in PLE. This indicated that pressure application did not have any effect on TPC, TFC and ABTS extraction, however pressure did reduce the extraction time. The present results agreed with the previous study showed that the PLE method reduced the extraction time in TPC extraction from passion fruit rinds by 12 times [18]. The PLE method also reduced the extraction time and the solvent use in polycyclic aromatic hydrocarbon (PAH) extraction from soil (18 and 10 times lower than Soxhlet extraction method, respectively) [19]. The extraction of phenolic compounds from *Mentha piperita*, *Origanum vulgare*, *Rosmarinus officinalis* L. and *Thymusvulgaris* L. by PLE method also gave a higher yield of phenolic and volatile compounds as well as the higher antioxidant activity compared to Soxhlet extraction method [20]. This did indicate the potential of PLE on MBC extraction.

### 2.2. Model Fitting and Analysis of Variance

Optimization of extraction conditions was performed in 45 randomized runs to study the effect of different variables on the TPC, TFC and ABTS of the MBC extract. The coded values, uncoded values and the results of dependent variables were shown in Table 1. Phenolic compounds were extracted from MBC ranged from 3.34 ± 0.32 to 52.88 ± 2.48 mg GAE/g MBC (dry weight, d.w.). Extracted flavonoids ranged from 1.27 ± 0.26 to 17.07 ± 0.53 mg CE/g MBC d.w. and ABTS ranged from 3.34 ± 0.80 to 202.71 ± 6.26 mg TE/g MBC d.w. These results showed that TPC, TFC and ABTS considerably depended on the extraction condition, which indicated that the optimization of the extraction condition was important. The optimization of the extraction process was performed by applying second-order polynomial equations. The regression coefficients for dependent variables along with the corresponding coefficient of determination value (R^2^) and lack of fit test of TPC, TFC and ABTS were shown in Table 2.

The ANOVA results indicated that three independent variables on the model were able to explain the experimental variation for TPC, TFC and ABTS as shown by the significant *p*-value for each model (Table 2). The corresponding coefficients of determination (R^2^) of the model were 0.9426, 0.9507 and 0.9809 for TPC, TFC and ABTS, respectively. These values implied that more than 94.26% of the response variables variation could be explained by the regression models. Although the lack of fit was significant, the correlation between predicted values and actual values of all runs was high (R^2^ = 0.9740, 0.8486 and 0.9877) for TPC, TFC and ABTS respectively (data was not shown). The predicted second-order polynomial regression equations with enter elimination method were shown in Table 3 for TPC, TFC and ABTS (Equations (1)–(3), respectively).

### 2.3. Effect of the Extraction Variables on TPC, TFC and ABTS

The model showed a high significant value with the experimental data. ANOVA showed a positive linear effect of temperature (X_1_) and ethanol concentration (X_3_) was significant for TPC. The linear effect of ethanol concentration (X_3_) was significant for ABTS, while all dependent variables, temperature (X_1_), pressure (X_2_) and ethanol concentration (X_3_) were significant for TFC. The quadratic effect of temperature (X_1_), pressure (X_2_) and ethanol concentration (X_3_) were found to be significant for TFC, while the quadratic effect of only ethanol concentration (X_3_) was found to be significant for TPC and ABTS. The interaction effect of temperature (X_1_) and ethanol concentration (X_3_) was found to be significant for TPC, while the interaction effect of temperature (X_1_) and pressure (X_2_) was found to be significant for TFC. There was no interaction effect of dependent variables on ABTS.

**Figure 2:**
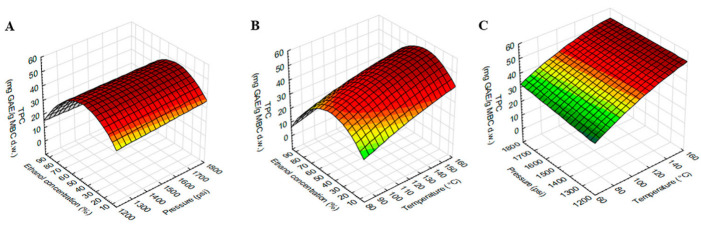
Response surface plots showing the interaction effects of extraction variables on TPC of MBC extract. (**A**) pressure and ethanol concentration at constant temperature (120 °C), (**B**) temperature and ethanol concentration at constant pressure 1500 psi and (**C**) temperature and pressure at constant ethanol concentration (50%).

The response surface plot of TPC using different combinations of temperature, pressure and ethanol concentration was shown in Figure 2. The TPC was higher at higher temperatures and vice versa, while pressure did not affect TPC. As shown in Table 2, TPC was significantly influenced by the interaction effect of temperature and ethanol concentration. Figure 2B showed that TPC increased with ethanol concentration increased, but this increase was up to 50% ethanol and then gradually decreased as ethanol concentration increased.

**Figure 3:**
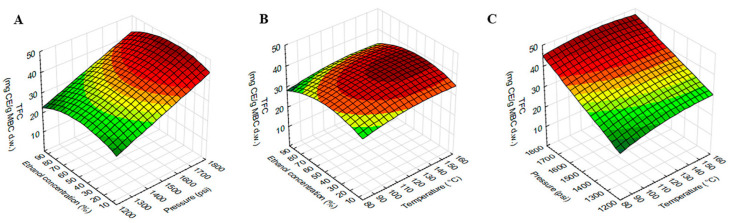
Response surface plots showing the interaction effects of extraction variables on TFC of MBC extract. (**A**) pressure and ethanol concentration at constant temperature (120 °C), (**B**) temperature and ethanol concentration at constant pressure 1500 psi and (**C**) temperature and pressure at constant ethanol concentration (50%).

The response surface plot of TFC using different combinations of temperature, pressure and ethanol concentration was shown in Figure 3. The TFC was higher at higher temperatures, pressure and vice versa. As shown in Table 2, TFC was significantly influenced by the interaction effect of temperature and pressure. The effect of temperature on TFC extraction seen in Figure 3C was positive but clearly lower than the effect of pressure. The effect of ethanol concentration on TFC was similar to TPC but less potent.

**Figure 4:**
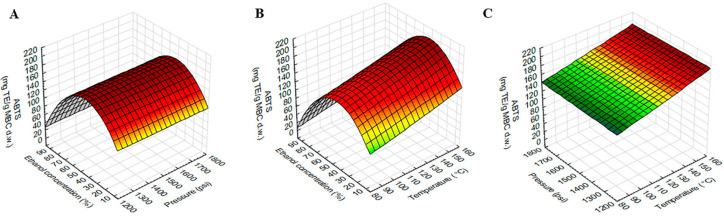
Response surface plots showing the interaction effects of extraction variables on ABTS of MBC extract. (**A**) pressure and ethanol concentration at constant temperature (120 °C), (**B**) temperature and ethanol concentration at constant pressure 1500 psi and (**C**) temperature and pressure at constant ethanol concentration (50%).

The response surface plot of ABTS using different combinations of temperature, pressure and ethanol concentration was shown in Figure 4. Figure 4A,B showed that ABTS increased with ethanol concentration increased, but this increase was up to 50% ethanol and then gradually decreased as ethanol concentration increased (>50%).

The present results were consistent with the previous studies showing that higher extraction temperature promoted higher phenolic compounds extracted from rosemary [21], jabuticaba skins [22], cocoa bean shell [23] and rice grains [16] using PLE. The use of high temperature promotes the recovery of phenolic and flavonoid compounds because it increases the molecular motion, which in turn causes a decrease of solvent viscosity, increased diffusivity and increased solubility of target compounds. High temperature also assists in making the cell walls more permeable, enabling the target compounds to leach out into the solvent. In addition, high temperature assists in breaking down target compound-matrix interaction and promote the diffusion of the target compound to the matrix surface and mass transfer to solvent [16,24].

Although the use of elevated temperature promotes higher phenolic compounds recovery, the use of excessive extraction temperature may degrade phenolic compounds. The use of elevated temperature in potato peel extraction also increased phenolic compounds recovery, but further temperature to 190 °C resulted in a decrease in phenolic compounds yield [25]. Another study reported that the increase of temperature to a certain level (190 °C) did not affect the level of phenolic compounds [16]. The positive effect of temperature on antioxidant activity was also found in the papaya seed extraction [26]. A similar trend of TPC and ABTS in the present study was due to the highly correlated between the content of phenolic compounds and antioxidant activity as shown in other study [27].

Pressure plays an important role in PLE extraction because at high temperatures, the solvent becomes a gas state. High pressure in PLE allows the solvent to maintain a liquid state, which increases the penetration of the solvent in the sample matrix [24]. The present results indicated that pressure did not significantly affect TPC and ABTS in MBC extraction. This may be due to the lowest pressure in the present study (1200 psi) being enough to maintain the ethanol mixture in a liquid state. Therefore, the increase of pressure did not have any effect on extraction. The insignificant effect of pressure in the present study was consistent with a previous extraction study in the potato peel [25]. However, pressure showed a significant effect on TFC in the present study. Application of pressure in PLE alone without high temperature increases the viscosity of the solvent and solvents with high viscosity result in slow mass transfer, lower diffusivity and lower extraction efficacy [28]. Therefore, both high temperature and pressure need to be applied together in PLE.

Ethanol was used in the present study since it is relatively safer or less toxic. The concentration of ethanol also showed an important effect on the TPC, TFC and ABTS. The present study found that the optimum ethanol concentration for MBC extraction was 50% ethanol. The lower and higher ethanol concentration than 50% showed the lower responses, especially TPC and ABTS. The optimum ethanol concentration in the present study was consistent with previous studies. Zafari and Sharifi reported that the mixture of alcohol with 50% water gave the highest flavonoids including vitexin from *Prosopis farcta* [29]. Sixty percent of ethanol was the best concentration to extract phenolic compounds from brewers’ spent grain compared to 20, 40 and 100% ethanol [30]. The vitexin and orientin yield from *Trollius chinesis* flowers extraction increased with the increase of ethanol concentration in the range of 0-60%. The highest yield was given at 60% ethanol extraction [31]. However, the yield sharply decreased when the ethanol concentration increased further. The present study and previous studies confirmed that ethanol concentration greatly impacted the phenolics and flavonoids extraction. Phenolic and flavonoid compounds are diverse with a wide range of solubilities in a single component [30]. Solvents with high polarity showed a higher ability to extract compounds with a wider polarity. Moreover, this allowed non-phenolic polar compounds such as protein and carbohydrates to dissolve during the extraction process, leading to the increased extraction yield [32]. This phenomenon was also explained by the fact that the addition of ethanol reduced the dielectric constant of extraction solvent, which facilitates the solubility and diffusion of phenolic compounds. Too high ethanol concentration, however, results in cell dehydration and denaturation of cell wall proteins, which inhibits the diffusion of phenolic compounds from plant material to extraction solvent [31]. Therefore, the mixture of ethanol and water was suitable for phenolic and flavonoid compounds extraction.

### 2.4. Optimization and Validation of the Extraction Condition

Multiple numerical optimizations were carried out to achieve the overall optimum PLE extraction conditions [33]. The goal was to set the optimum level of independent variables resulting in the highest TPC, TFC and ABTS. The predicted optimum extraction conditions from Minitab were: temperature of 160 °C, pressure of 1300 psi and 35% ethanol. Under recommended optimum extraction conditions, the predicted and experimental values provided residual standard error (RSE) of more than 10% (data was not shown). This indicated that the recommended optimum extraction conditions were not the actual optimum, which was indicated by the significant lack of fit of the models. Therefore, the steepest ascent method was carried out to find the actual optimum extraction condition. The procedure of the steepest ascend method is moving sequentially in the direction of the maximum response and based on the previous experiment [34]. According to Table 1, the conditions of temperature of 160 °C, pressure of 1200 psi and 50% ethanol provided the highest TPC, TFC and ABTS compared to other treatments. Therefore, 50% ethanol was selected to be a new optimum ethanol concentration. The new optimum conditions were temperature of 160 °C, pressure of 1300 psi and 50% ethanol. Under the new optimum extraction conditions, the predicted TPC, TFC and ABTS were 53.28 GAE/g MBC d.w., 32.88 mg CE/g MBS d.w. and 192.20 mg TE/g MBC d.w., respectively (Table 4). MBC was extracted under the new optimum conditions to verify the validity. The TPC, TFC and ABTS of the extract were then determined to verify the reliability of the new optimum conditions. The validation study showed the TPC, TFC and ABTS of the new MBC extracted solution were 55.267 ± 1.14 mg GAE/g MBC d.w., 34.041 ± 0.72 mg CE/g MBS d.w. and 195.046 ± 2.29 mg TE/g MBC d.w., respectively (Table 4). Under the new optimum extraction conditions, the experimental values showed that the models were in good agreement with the predicted values with RSE values of less than 4%. The present result confirmed the reliability of the new optimum extraction conditions.

### 2.5. Scale-Up Study

**Figure 5:**
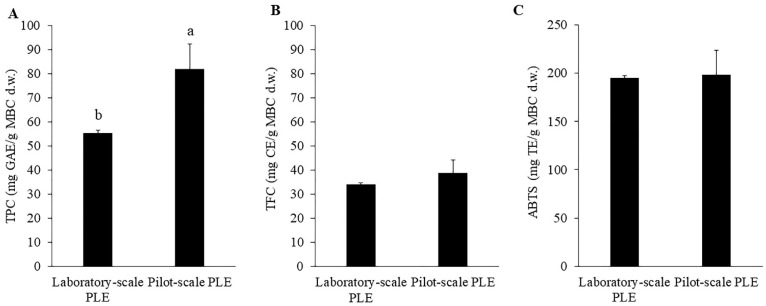
Total phenolic content (**A**), total flavonoid content (**B**) and ABTS (**C**) of MBC extract obtained by laboratory-scale PLE and pilot-scale PLE. There was no significant difference between laboratory-scale PLE and pilot-scale PLE. The different alphabet indicated significant difference at *p* < 0.05.

One of the main goals of this study was to scale up the extraction process of MBC. Many factors influence the upscaling extraction process such as process parameters, the kinetics of the whole process, the ratio between material and solvent [22]. The pilot-scale PLE (10 L extraction cell) was 25 times bigger than the laboratory-scale PLE (400 mL extraction cell). The optimum extraction conditions were used in the pilot-scale extraction with the same solid to solvent ratio (1:5 *w*/*v*; 2 kg MBC in 10 L ethanol). The extraction time was constant with laboratory-scale PLE for 10 min. Comparing TPC, TFC and ABTS of the laboratory-scale PLE and pilot-scale PLE (Figure 5), the results showed that TFC and ABTS from pilot-scale PLE were not significantly different from TFC and ABTS from laboratory-scale PLE. Interestingly, TPC from pilot-scale PLE was significantly higher than that from laboratory-scale PLE. The increase of TPC might be due to the change of geometric factors of laboratory-scale PLE and pilot-scale PLE. Previous study showed that geometry and dynamic factors influenced the scale-up study [35]. However, this increase should be more investigated for the explanation. These results indicated that MBC extraction with pilot-scale PLE was efficiently comparable to laboratory-scale PLE.

### 2.6. Identification of Vitexin and Isovitexin in the Extract

**Figure 6:**
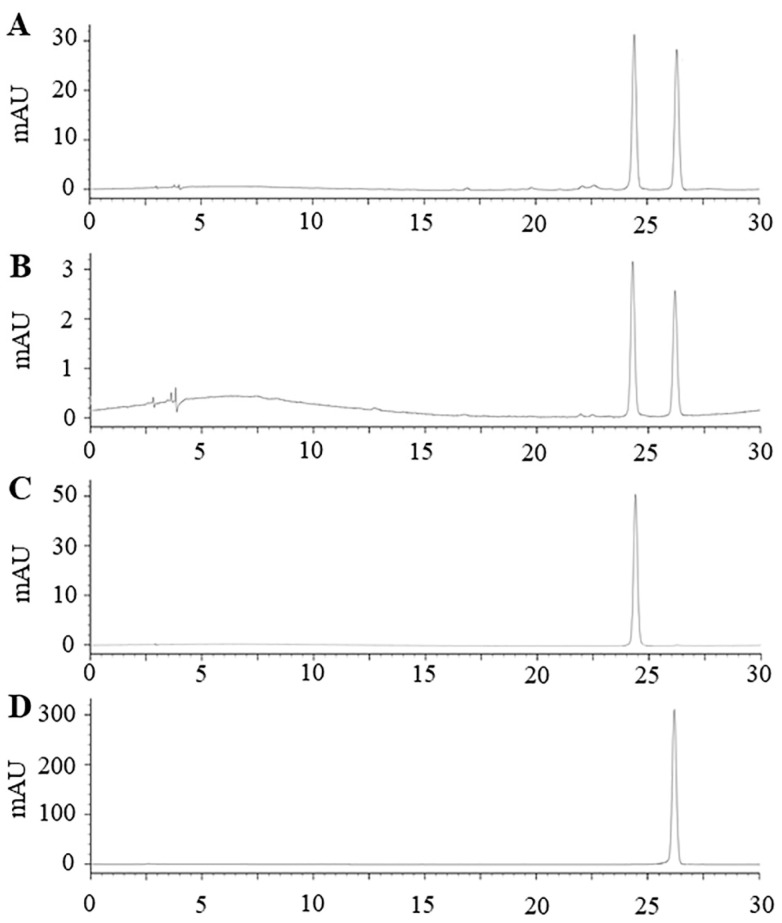
Representative high performance liquid chromatography-diode array detector chromatographic profile (λ = 337 nm) of (**A**) mung bean seed coat extract from laboratory scale; (**B**) mung bean seed coat freeze-dried extract from pilot scale; (**C**) standard vitexin; (**D**) standard isovitexin.

Vitexin and isovitexin are major flavonoids found in mung beans especially in seed coat [4]. Our previous study reported that mung bean seed coat extracted using boiling water contained vitexin and isovitexin 38.56 and 28.96 mg/g extract [5]. The present study showed that mung bean seed coat extracted using PLE contain both vitexin and isovitexin at the concentration of 130.53 ± 17.89 and 21.21 ± 3.22 mg/g freeze-dried extract (Figure 6).

In the present study, TPC and TFC of MBC extracted with the optimum conditions using scale-up PLE were 81.88 mg GAE/g MBC d.w. and 38.90 mg CE/g MBC d.w., respectively. TPC and TFC from the present study were higher than TPC and TFC of MBC extract obtained from maceration extraction method (29.58 mg GAE/g MBC and 22.08 mg CE/g MBC, respectively) [4]. UAE was also applied to increase TPC and TFC extraction. However, TPC and TFC from MBC extract obtained from UAE were only 42.22 mg GAE/g MBC and 1.13 mg CE/g MBC, respectively [9]. This indicated that PLE was a potential extraction method to extract phenolic and flavonoid compounds from MBC with high efficiency.

## 3. Materials and Methods

### 3.1. Materials and Chemicals

Mung bean seed coat (MBC), a co-product of the mung bean dehulled process, was received from Kittitat Co., Ltd. (Bang Khun Thian, Bangkok, Thailand) in May 2019. After foreign matters removal, MBC was kept in sealed plastic bags at 4 °C until extraction. Ethanol commercial grade (Mallinckrodt Baker Inc., Phillipsburg, NI, USA) was used for extraction. ABTS, vitexin and isovitexin were purchased from Sigma-Aldrich (St. Louis, MO, USA). All other chemicals were of analytical grade and obtained from reputable suppliers.

### 3.2. Extraction Procedures

#### 3.2.1. Soxhlet Extraction

MBC was extracted by Soxhlet extraction according to Weggler et al. [36]. Briefly, 8 g of MBC was placed in an extraction thimble. The thimble was then placed in a Soxhlet extractor and 400 mL of 95% ethanol was used as a solvent. The extraction was carried out for 6 h at 80 °C. The extract was collected in a Duran bottle and kept in a dark place at 4 °C. The extract was centrifuged at 7000 rpm, room temperature for 10 min. The supernatant was collected in a Duran bottle and kept in a dark place at 4 °C until further analysis.

#### 3.2.2. Pressurized Liquid Extraction (PLE) at Laboratory Scale

**Figure 7:**
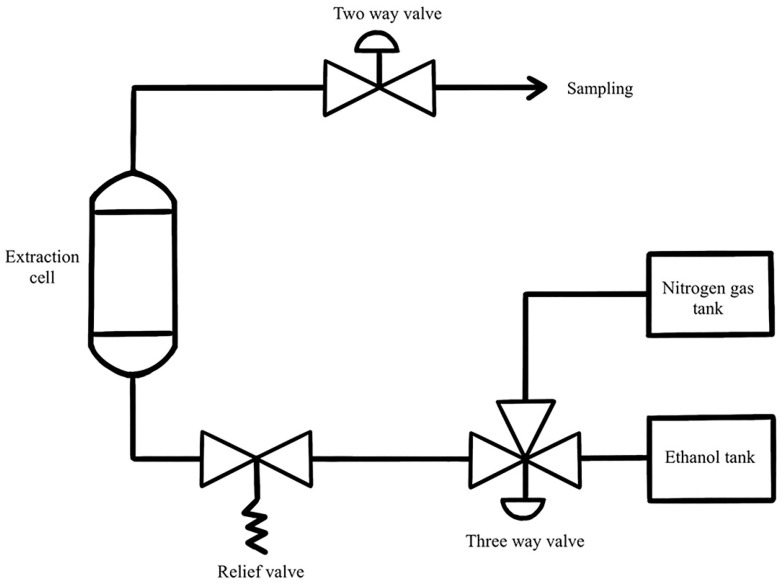
Pressurized liquid extraction set-up.

PLE unit was assembled in the Chemical Engineering Laboratory, Department of Chemical Engineering, Faculty of Engineering, Kasetsart University, Bangkok, Thailand (Figure 7). The solvent was pumped by a preparative pump (Fluid Management System, Kalamazoo, MI, USA) into the extraction cell. The extraction cell was placed between an electrical heating system at the desired temperature until the required pressure was obtained. All connections within the system were made using stainless steel tubes.

MBC was placed in the 400 mL extraction cell (OD 60.33 mm, length 28 mm, stainless steel 316) containing a metal filter at the top and bottom of the extraction cell. The cell containing the sample was filled with extraction solvent, heated and then pressurized. The sample was placed in the heating system for 10 min at desired temperature and pressure. After 10 min, the relief valve was carefully opened to lower pressure to atmospheric pressure. After temperature was below 70 °C, the cell was then exhaustively purged with 350 mL 50% ethanol to ensure that no residual extract solution was left in the extraction cell. The MBC extract was collected in a Duran bottle and kept in a dark place at 4 °C. The MBC extract was centrifuged at 7000 rpm, room temperature for 10 min. The supernatant was collected in a Duran bottle and kept in a dark place at 4 °C until further analysis.

#### 3.2.3. Pressurized Liquid Extraction (PLE) at Pilot Scale

In order to scale up the extraction process, the pilot-scale PLE was used. This PLE was equipped with a 10L extraction cell (OD 143.2 mm, length 1200 mm, stainless steel 316). The optimum conditions for extraction were repeated at the pilot scale. The difference was that 2 kg of MBC was used in the extraction cell. After 10 min extraction, the MBC extract was collected in a plastic bottle and kept in a dark place at 4 °C. The MBC extract was centrifuged at 7000 rpm, room temperature for 10 min. The supernatant was collected and evaporated to remove the solvent at 60 °C, then the sample was freeze-dried and kept in an aluminum foil bag at 4 °C for further analysis.

### 3.3. Extract Characterization

#### 3.3.1. Determination of Total Phenolic Content (TPC)

TPC was analyzed according to Herald et al. [37]. Briefly, 75 µL of distilled water was added to each well of the 96-well plate, followed by 25 µL of sample and 25 µL of folin ciocalteu reagent (diluted 1:1 (*v*/*v*) with distilled water). After the solutions had been mixed and left for 6 min, then 100 µL of 75 g/L Na_2_CO_3_ was added to each well. The solutions were mixed again, and the plates were covered and left at room temperature in a dark place for 90 min. After shaking for 60 sec, the absorbance was measured at 765 nm using a microplate reader (TECAN, Infinite 200 Pro, Manndenorf, Switzerland). Gallic acid was used as a reference standard and the results were expressed as mg GAE/g MBC (d.w.).

#### 3.3.2. Determination of Total Flavonoid Content (TFC)

TFC was analyzed according to Herald et al. [37]. Briefly, 100 µL of distilled water was added to each well of the 96-well plate, followed by 10 µL of 50 g/L NaNO_2_ and 25 µL of sample solution. After 5 min incubation, 15 µL of 100 g/L AlCl_3_ was added to the mixture. Six min later, 50 µL of 1 M NaOH and 50 µL of distilled water were added. The plate was shaken for 30 sec in the plate reader before measuring absorbance at 510 nm using a microplate reader (TECAN, Infinite 200 Pro, Manndenorf, Switzerland). Catechin was used as a reference standard and the results were expressed as mg CE/g MBC (d.w.).

#### 3.3.3. Determination of ABTS Radical Scavenging Activity (ABTS)

The 2,2′-azinobis(3-ethylbenzothiazoline-6-sulfonic acid) (ABTS) radical scavenging activity was determined according to Indracanti et al. [38]. Briefly, 10 µL of sample were mixed with 190 µL of ABTS solution (2.45 mM potassium persulfate solution and 7 mM ABTS). The mixture was incubated at room temperature for 6 min in a dark place, then the absorbance was measured at 734 nm using a microplate reader (TECAN, Infinite 200 Pro, Manndenorf, Switzerland). Trolox was used as a reference standard and the results were expressed as mg TE/g MBC (d.w.).

### 3.4. Identification of Major Compounds in the Extract

The major compounds in the extract were determined using high-performance liquid chromatography (HPLC) according to our previous method [39]. The extract was dissolved with deionized water and subjected to HPLC equipped with a diode array detector (Waters 600, Milford, MA, USA). An analytical column (C18) (4.6 × 250 mm, Inertsil ODS-3, 5 µm, GL Sciences, Tokyo, Japan) was used and kept at 30 °C while using. The spectra from 210–600 nm were recorded and UV absorbance at 337 nm was used to monitor flavonoids. The injection volume was 10 µL at a flow rate of 1 mL/min. Elution was done using two solvent gradients: solvent A (1% acetic acid in deionized water) and solvent B (1% acetic acid in methanol). The gradient program started with 10–35% B (10 min), 35–42% B (15 min), 42–75% B (10 min), 75% B (5 min), 75–10% B (5 min) and 10% B (5 min). Vitexin and isovitexin at the concentrations 20, 40, 60, 80 and 100 mg/kg were used as external standards to make standard curves and to determine the concentration of vitexin and isovitexin in the extract.

### 3.5. Experimental Design and Statistical Analysis

The effect of three independent variables: temperature (X_1_; 80–160 °C), pressure (X_2_; 1200–1800 psi) and ethanol concentration (X_3_; 5–95%) on the response variables: TPC (Y_1_), TFC (Y_2_) and ABTS (Y_3_) was evaluated using a three-factor-three-level Box-Behnken design (BBD). The three coded levels of temperature, pressure and ethanol concentration were incorporated into the design and were analyzed in 15 combinations (Table 1). The central point of the design was repeated three times to estimate the repeatability of the method. For each combination, three dependent variables were determined. A second-order polynomial model was used for fitting data and predicting the responses as Equation (4):

(4) $$Y=b_{0}+b_{1}X_{1}+b_{2}X_{2}+b_{3}X_{3}+b_{11}X_{1}^{2}+b_{22}X_{2}^{2}+b_{33}X_{3}^{2}+b_{12}X_{1}X_{2}+b_{13}X_{1}X_{3}+b_{23}X_{2}X_{3}$$

where, *Y* is the response variable; $b_{0}$, $b_{1}$, $b_{2}$, $b_{3}$, $b_{11}$, $b_{22}$, … are regression coefficients and $X_{1}$, $X_{2}$ and $X_{3}$ are uncoded values for temperature, pressure and ethanol concentration, respectively. An analysis of variance (ANOVA) was performed at a 95% confidence level to evaluate the predicted model on the response variables and assess the effect of each factor. In addition, the regression coefficient (R^2^), the *p*-value of the regression model, the *p*-value of the lack of fit were used to determine the fitness of the regression model. The graphical and numerical optimization procedures were used to determine the optimum PLE condition. Three-dimensional (3D) response surface plots were applied for the interactions. Steepest ascend approach was also applied to determine the optimum PLE conditions to provide the highest TPC, TFC and ABTS. In order to assess the adequacy of the constructed model, the actual values were compared with the predicted values and the percentage of the residual standard error (RSE) was calculated for each response [40]. All of the treatments were carried out in triplicates. The experimental design matrix, data analysis, regression coefficients and numerical optimization were analyzed using Minitab statistical software (Trial version 16.1 Minitab Inc., State College, PA, USA) and 3D graphs were provided using Statistica 10.0 (StatSoft Inc., Tulsa, OK, USA). Differences between groups of data were assessed by student t-test. Results were expressed as mean ± standard deviation.

## 4. Conclusions

The present study showed the possibility of obtaining phenolics, flavonoids with antioxidant activity from MBC using PLE. The effects of temperature, pressure and ethanol concentration were studied to maximize TPC, TFC and ABTS of MBC extract. The results showed that TPC, TFC and ABTS were most affected by ethanol concentration. TPC and TFC were also affected by temperature, while pressure did not affect TPC and ABTS. Temperature and ethanol concentration had an interaction effect on TPC, while temperature and pressure had an interaction effect on TFC. The optimum conditions obtained from numerical optimization and steepest ascend approach, were temperature of 160 °C, pressure of 1300 psi and 50% ethanol. The optimum conditions were performed in laboratory-scale and pilot-scale PLE. No significant decrease was observed in pilot-scale PLE. The profile of MBC extract from laboratory-scale and pilot-scale PLE also remained. This illustrated that the optimum conditions can be transferred to an industrial scale. However, industrial costs such as energy and materials are needed to be investigated. In conclusion, the PLE process appeared to be a potential technique in extracting phenolics, flavonoids with antioxidant activity from mung bean seed coat.

## Figures and Tables

**Table 1:** Coded and uncoded levels of independent variables in experimental design, TPC, TFC and ABTS from the MBC extract.

Treatment	Temperature (°C)		Pressure (psi)		Ethanol Concentration (%)		TPC (mg GAE/g MBC d.w.)	TFC (mg CE/g MBC d.w.)	ABTS (mg TE/g MBC d.w.)
	Code	Uncode	Code	Uncode	Code	Uncode			
1	−1	80	0	1500	+1	95	3.34 ± 0.32 ^e^	1.27 ± 0.26 ^g^	3.34 ± 0.80 ^g^
2	0	120	−1	1200	+1	95	6.43 ± 0.31 ^e^	4.93 ± 1.27 ^f^	9.80 ± 1.74 ^g^
3	0	120	+1	1800	+1	95	6.22 ± 0.39 ^e^	5.45 ± 0.81 ^e,f^	11.49 ± 1.67 ^g^
4	+1	160	0	1500	+1	95	6.73 ± 0.73 ^e^	6.73 ± 0.51 ^e,f^	36.70 ± 2.64 ^f^
5	−1	80	−1	1200	0	50	28.06 ± 2.11 ^c,d^	7.22 ± 0.67 ^e^	123.59 ± 5.90 ^c,d^
6	−1	80	+1	1800	0	50	30.78 ± 1.94 ^c^	6.80 ± 0.62 ^e,f^	133.74 ± 2.01 ^c^
7	+1	160	−1	1200	0	50	52.88 ± 2.48 ^a^	17.07 ± 0.53 ^a^	197.51 ± 7.99 ^a^
8	0	120	0	1500	0	50	36.91 ± 1.63 ^b^	14.13 ± 0.43 ^b^	155.29 ± 9.98 ^b^
9	0	120	0	1500	0	50	38.81 ± 2.40 ^b^	14.21 ± 0.82 ^b^	163.50 ± 9.40 ^b^
10	0	120	0	1500	0	50	37.97 ± 3.74 ^b^	13.90 ± 0.98 ^b^	169.08 ± 8.90 ^b^
11	+1	160	+1	1800	0	50	50.94 ± 0.53 ^a^	13.13 ± 0.45 ^b,c^	202.71 ± 6.26 ^a^
12	−1	80	0	1500	−1	5	23.47 ± 0.44 ^d^	7.26 ± 0.45 ^e^	87.58 ± 0.62 ^e^
13	0	120	−1	1200	−1	5	29.85 ± 2.72 ^c^	9.76 ± 0.34 ^d^	110.07 ± 2.07 ^d^
14	0	120	+1	1800	−1	5	36.32 ± 1.22 ^b^	11.81 ± 0.44 ^c,d^	118.15 ± 1.29 ^c,d^
15	+1	160	0	1500	−1	5	37.61 ± 0.80 ^b^	12.96 ± 0.90 ^b,c^	124.35 ± 1.52 ^c,d^

Means values with the different letters in the same column are significantly different (*p* ≤ 0.05).

**Table 2:** Regression coefficients, coefficient of determination and *p*-value of the predicted second-order polynomial models for TPC, TFC and ABTS.

Regression Term	TPC		TFC		ABTS	
	Coefficient	*p*-Value *	Coefficient	*p*-Value *	Coefficient	*p*-Value *
Constant	−51.7964	0.225	−57.4491	0.000	−10.0851	0.916
Temperature (X_1_)	0.7024	0.011	0.4937	0.000	0.6657	0.270
Pressure (X_2_)	0.0206	0.664	0.0443	0.000	0.0097	0.928
Ethanol (X_3_)	1.2036	0.000	0.2303	0.000	4.1609	0.000
Temperature × Temperature (X_1_X_1_)	−0.0012	0.164	−0.0012	0.000	0.0007	0.699
Pressure × Pressure (X_2_X_2_)	0.0000	0.998	0.0000	0.003	0.0000	0.852
Ethanol × Ethanol (X_3_X_3_)	−0.0113	0.000	−0.0025	0.000	−0.0498	0.000
Temperature × Pressure (X_1_X_2_)	−0.0001	0.374	−0.0001	0.009	−0.0001	0.675
Temperature × Ethanol (X_1_X_3_)	−0.0015	0.045	0.0000	0.847	−0.0005	0.773
Pressure × Ethanol (X_2_X_3_)	−0.0001	0.205	0.0000	0.240	−0.0001	0.589
R^2^	0.9426	-	0.9507	-	0.9809	-
R^2^ adjusted	0.9279	-	0.9380	-	0.9760	-
Regression	-	0.000	-	0.000	-	0.000
Lack of fit	-	0.000	-	0.000	-	0.000

* The *p*-value more than 0.05 is not significantly different at the 5% level.

**Table 3:** Predicted second-order polynomial model equations of TPC, TFC and ABTS.

Responses	Polynomial Equations	
TPC (mg GAE/g MBC d.w.)	$Y=51.7964+0.7024X_{1}+0.0206X_{2}+1.2036X_{3}-0.0012X_{1}^{2}-0.0113X_{3}^{2}\;-0.0001X_{1}X_{2}-0.0015X_{1}X_{3}-0.0001X_{2}X_{3}\;$	(1)
TFC (mg CE/g MBC d.w.)	$Y=57.4491+0.4937X_{1}+0.0443X_{2}+0.2303X_{3}-0.0012X_{1}^{2}-0.0025X_{3}^{2}\;-0.0001X_{1}X_{2}$	(2)
ABTS (mg TE/g MBC d.w.)	$Y=10.0851+0.6657X_{1}+0.0097X_{2}+4.1609X_{3}+0.0007X_{1}^{2}-0.0498X_{3}^{2}\;-0.0001X_{1}X_{2}-0.0005X_{1}X_{3}-0.0001X_{2}X_{3}$	(3)

**Table 4:** Predicted and experimental response values at the optimum extraction conditions.

Response Variables	Predicted Values	Experimental Values	RSE (%)
TPC (mg GAE/g MBC d.w.)	53.28	55.27 ± 1.14 ns	3.73
TFC (mg CE/g MBC d.w.)	32.88	34.04 ± 0.72 ns	3.54
ABTS (mg TE/g MBC d.w.)	192.20	195.05 ± 2.29 ns	1.48

## Data Availability

The dataset generated for this research are available on request to the corresponding author.

## Sample Availability

Samples of the compounds are not available from the authors.

## References

[1] Yi-Shen Z., Shuai S., Fitzgerald R. (2018). Mung bean proteins and peptides: Nutritional, functional and bioactive properties. Food Nutr. Res..

[2] Anwar F., Latif S., Przybylski R., Sultana B., Ashraf M. (2007). Chemical Composition and Antioxidant Activity of Seeds of Different Cultivars of Mungbean. J. Food Sci..

[3] Hou D., Yousaf L., Xue Y., Hu J., Wu J., Hu X., Feng N., Shen Q. (2019). Mung Bean (*Vigna radiata* L.): Bioactive Polyphenols, Polysaccharides, Peptides, and Health Benefits. Nutrients.

[4] Luo J., Cai W., Wu T., Xu B. (2016). Phytochemical distribution in hull and cotyledon of adzuki bean (*Vigna angularis* L.) and mung bean (*Vigna radiate* L.), and their contribution to antioxidant, anti-inflammatory and anti-diabetic activities. Food Chem..

[5] Sae-tan S., Kumrungsee T., Yanaka N. (2020). Mungbean seed coat water extract inhibits inflammation in LPS-induced acute liver injury mice and LPS-stimulated RAW 246.7 macrophages via the inhibition of TAK1/IκBα/NF-κB. J. Food Sci. Technol..

[6] Cao D., Li H., Yi J., Zhang J., Che H., Cao J., Yang L., Zhu C., Jiang W. (2011). Antioxidant properties of the mung bean flavonoids on alleviating heat stress. PLoS ONE.

[7] Jang Y.-H., Kang M.-J., Choe E.-O., Shin M., Kim J.-I. (2014). Mung bean coat ameliorates hyperglycemia and the antioxidant status in type 2 diabetic db/db mice. Food Sci. Biotechnol..

[8] Zhao Y., Du S.-K., Wang H., Cai M. (2014). In vitro antioxidant activity of extracts from common legumes. Food Chem..

[9] Singh B., Singh N., Thakur S., Kaur A. (2016). Ultrasound assisted extraction of polyphenols and their distribution in whole mung bean, hull and cotyledon. J. Food Sci. Technol..

[10] Lee J.H., Jeon J.K., Kim S.G., Kim S.H., Chun T., Imm J.-Y. (2011). Comparative analyses of total phenols, flavonoids, saponins and antioxidant activity in yellow soy beans and mung beans. Int. J. Food Sci. Technol..

[11] Oreopoulou A., Tsimogiannis D., Oreopoulou V., Watson R.R. (2019). Chapter 15—Extraction of Polyphenols from Aromatic and Medicinal Plants: An Overview of the Methods and the Effect of Extraction Parameters. Polyphenols in Plants.

[12] Santos K.A., Gonçalves J.E., Cardozo-Filho L., da Silva E.A. (2019). Pressurized liquid and ultrasound-assisted extraction of α-bisabolol from candeia (*Eremanthus erythropappus*) wood. Ind. Crops Prod..

[13] Machado A.P.D.F., Pereira A.L.D., Barbero G.F., Martínez J. (2017). Recovery of anthocyanins from residues of *Rubus fruticosus*, *Vaccinium myrtillus* and *Eugenia brasiliensis* by ultrasound assisted extraction, pressurized liquid extraction and their combination. Food Chem..

[14] Péres V.F., Saffi J., Melecchi M.I.S., Abad F.C., de Assis Jacques R., Martinez M.M., Oliveira E.C., Caramão E.B. (2006). Comparison of soxhlet, ultrasound-assisted and pressurized liquid extraction of terpenes, fatty acids and Vitamin E from Piper gaudichaudianum Kunth. J. Chromatogr. A.

[15] Barriada-Pereira M., González-Castro M.J., Muniategui-Lorenzo S., López-Mahía P., Prada-Rodríguez D., Fernández-Fernández E. (2007). Comparison of pressurized liquid extraction and microwave assisted extraction for the determination of organochlorine pesticides in vegetables. Talanta.

[16] Setyaningsih W., Saputro I., Palma M., Barroso C. (2016). Pressurized liquid extraction of phenolic compounds from rice (*Oryza sativa*) grains. Food Chem..

[17] Mandal S.C., Mandal V., Das A.K., Mandal S.C., Mandal V., Das A.K. (2015). Chapter 6—Classification of Extraction Methods. Essentials of Botanical Extraction.

[18] Viganó J., Brumer I.Z., de Campos Braga P.A., da Silva J.K., Maróstica Júnior M.R., Reyes Reyes F.G., Martínez J. (2016). Pressurized liquids extraction as an alternative process to readily obtain bioactive compounds from passion fruit rinds. Food Bioprod. Process..

[19] Hawthorne S.B., Grabanski C.B., Martin E., Miller D.J. (2000). Comparisons of Soxhlet extraction, pressurized liquid extraction, supercritical fluid extraction and subcritical water extraction for environmental solids: Recovery, selectivity and effects on sample matrix. J. Chromatogr. A.

[20] Rodríguez-Solana R., Salgado J.M., Domínguez J.M., Cortés-Diéguez S. (2015). Comparison of Soxhlet, accelerated solvent and supercritical fluid extraction techniques for volatile (GC-MS and GC/FID) and phenolic compounds (HPLC-ESI/MS/MS) from *Lamiaceae* species. Phytochem. Anal.

[21] Herrero M., Plaza M., Cifuentes A., Ibáñez E. (2010). Green processes for the extraction of bioactives from Rosemary: Chemical and functional characterization via ultra-performance liquid chromatography-tandem mass spectrometry and in-vitro assays. J. Chromatogr. A.

[22] Santos D.T., Veggi P.C., Meireles M.A.A. (2012). Optimization and economic evaluation of pressurized liquid extraction of phenolic compounds from jabuticaba skins. J. Food Eng..

[23] Okiyama D.C.G., Soares I.D., Cuevas M.S., Crevelin E.J., Moraes L.A.B., Melo M.P., Oliveira A.L., Rodrigues C.E.C. (2018). Pressurized liquid extraction of flavanols and alkaloids from cocoa bean shell using ethanol as solvent. Food Res. Int..

[24] De la Guardia M., Armenta S., Guardia M.D.L., Armenta S. (2011). Chapter 5—Greening Sample Treatments. Comprehensive Analytical Chemistry.

[25] Luthria D.L. (2012). Optimization of extraction of phenolic acids from a vegetable waste product using a pressurized liquid extractor. J. Funct. Foods.

[26] Samaram S., Mirhosseini H., Tan C.P., Ghazali H.M., Bordbar S., Serjouie A. (2015). Optimisation of ultrasound-assisted extraction of oil from papaya seed by response surface methodology: Oil recovery, radical scavenging antioxidant activity and oxidation stability. Food Chem..

[27] Wangkiri N., Sarnsri T., Thongkanjana T., Sae-tan S. (2021). Antioxidant potentials and inhibitory activities against α-amylase and α-glucosidase, and glucose uptake activity in insulin-resistance HepG2 cells of some medicinal plants. Agric. Nat. Resour..

[28] Poole C.F., Poole C.F. (2020). Chapter 2—Solvent Selection for Liquid-Phase Extraction. Liquid-Phase Extraction.

[29] Zafari S., Sharifi M. (2020). Optimization of Solvent Systems for the Extraction of Vitexin as the Major Bioactive Flavonoid in Prosopis farcta. Am. J. Plant Sci..

[30] Zuorro A., Iannone A., Lavecchia R. (2019). Water–Organic Solvent Extraction of Phenolic Antioxidants from Brewers’ Spent Grain. Processes.

[31] Chen F., Zhang Q., Liu J., Gu H., Yang L. (2017). An efficient approach for the extraction of orientin and vitexin from Trollius chinensis flowers using ultrasonic circulating technique. Ultrason. Sonochem..

[32] Ermi Hikmawanti N.P., Fatmawati S., Asri A.W. (2021). The Effect of Ethanol Concentrations as The Extraction Solvent on Antioxidant Activity of Katuk (*Sauropus androgynus* (L.) Merr.) Leaves Extracts. IOP Conf. Ser. Earth Environ. Sci..

[33] Mirhosseini H., Tan C.P., Taherian A.R., Boo H.C. (2009). Modeling the physicochemical properties of orange beverage emulsion as function of main emulsion components using response surface methodology. Carbohydr. Polym..

[34] Montgomery D.C. (2012). Design and Analysis of Experiments.

[35] Fernández-Ponce M.T., Parjikolaei B.R., Lari H.N., Casas L., Mantell C., de la Ossa E.J.M. (2016). Pilot-plant scale extraction of phenolic compounds from mango leaves using different green techniques: Kinetic and scale up study. Chem. Eng. J..

[36] Weggler B.A., Gruber B., Teehan P., Jaramillo R., Dorman F.L., Snow N.H. (2020). Chapter 5—Inlets and sampling. Separation Science and Technology.

[37] Herald T., Gadgil P., Tilley M. (2012). High-throughput micro plate assays for screening flavonoid content and DPPH-scavenging activity in sorghum bran and flour. J. Sci. Food Agric..

[38] Indracanti M., ChV S., Sisay T. (2019). A 96 well-microtiter plate abts based assay for estimation of antioxidant activity in green leafy vegetables. Biotechnol. Int..

[39] Saeting O., Chandarajoti K., Phongphisutthinan A., Hongsprabhas P., Sae-tan S. (2021). Water Extract of Mungbean (*Vigna radiata* L.) Inhibits Protein Tyrosine Phosphatase-1B in Insulin-Resistant HepG2 Cells. Molecules.

[40] Che Sulaiman I.S., Basri M., Fard Masoumi H.R., Chee W.J., Ashari S.E., Ismail M. (2017). Effects of temperature, time, and solvent ratio on the extraction of phenolic compounds and the anti-radical activity of Clinacanthus nutans Lindau leaves by response surface methodology. Chem. Cent. J..

